# Characterization of the Sublimation and Vapor Pressure of 2-(2-Nitrovinyl) Furan (G-0) Using Thermogravimetric Analysis: Effects of Complexation with Cyclodextrins

**DOI:** 10.3390/molecules200815175

**Published:** 2015-08-19

**Authors:** Vivian Ruz, Mirtha Mayra González, Danny Winant, Zenaida Rodríguez, Guy Van den Mooter

**Affiliations:** 1 Departamento de Farmacia, Facultad de Química y Farmacia, Carretera a Camajuaní km 5½, Universidad Central de Las Villas, 54830 Villa Clara, Cuba; E-Mails: vivianr@uclv.edu.cu (V.R.); mmayra@uclv.edu.cu (M.M.G.); 2 Department of Metallurgy and Material Engineering, Kasteelpark Arenberg 44, University of Leuven (KU Leuven), BE-3001 Heverlee, Belgium; E-Mail: danny.winant@mtm.kuleuven.be; 3 Centro de Bioactivos Químicos, Carretera a Camajuaní km 5½, Universidad Central de Las Villas, 54830 Villa Clara, Cuba; E-Mail: zrodríguez@uclv.edu.cu; 4 Drug Delivery and Disposition, Department of Pharmaceutical and Pharmacological Sciences, O & N2 Herestraat 49-Box 921, University of Leuven (KU Leuven), BE-3000 Leuven, Belgium

**Keywords:** 2-(2-nitrovinyl) furan, cyclodextrins, thermogravimetry, sublimation enthalpy, vapor pressure

## Abstract

In the present work, the sublimation of crystalline solid 2-(2-nitrovinyl) furan (G-0) in the temperature range of 35 to 60 °C (below the melting point of the drug) was studied using thermogravimetric analysis (TGA). The sublimated product was characterized using Fourier-transformed-infrared spectroscopy (FT-IR) and thin layer chromatography (TLC). The sublimation rate at each temperature was obtained using the slope of the linear regression model and followed apparent zero-order kinetics. The sublimation enthalpy from 35 to 60 °C was obtained from the Eyring equation. The Gückel method was used to estimate the sublimation rate and vapor pressure at 25 °C. Physical mixtures, kneaded and freeze-dried complexes were prepared with 2-hydroxypropyl-β-cyclodextrin (HP-β-CD) and sulfobutyl ether-β-cyclodextrin (SBE-β-CD) and analyzed using isothermal TGA at 50 °C. The complexation contributed to reducing the sublimation process. The best results were achieved using freeze-dried complexes with both cyclodextrins.

## 1. Introduction

**Figure 1:**
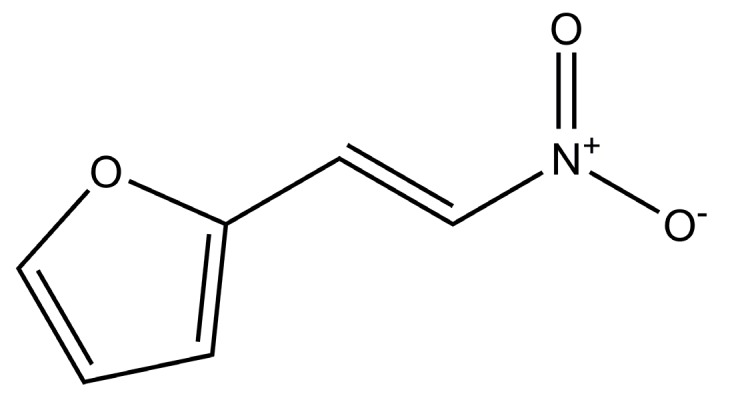
Chemical structure of 2-(2-nitrovinyl) furan (G-0).

2-(2-nitrovinyl) furan (G-0) (Figure 1) is an antiprotozoal agent, showing activity against *Eimeria tenella* (*in vitro* and *in vivo*) [1], *Trypanosoma cruzi* and *Leishmania* spp. (*in vitro*) [2]. More recently, it was demonstrated that it shows antimicrobial properties against pathogens like *Staphylococcus epidermidis*, *Shigella dysenteriae*, *Pseudomonas fluorescens* and *Candida albicans*, among others [3]. The drug is classified as non-genotoxic [4,5] and non-mutagenic [6], moderately toxic based on LD_50_ values obtained from three different experimental models and using two different routes of administration (oral and intraperitoneal) and a slight irritant of the skin and eyes [1,2].

This compound is also used as starting material for the manufacture of the bromide derivative 2-bromo-5-(2-bromo-2-nitrovinyl)-furan (G-1), which finds its application in the pharmaceutical and biotechnological industry due to its broad spectrum as an antifungal and antibacterial product.

Nitro compounds have the tendency to suffer from sublimation [7]. During the preformulation studies, G-0 showed some signals of sublimation, evidenced in weight loss when the powder was stored in unclosed containers at room temperature and when white plastic caps were used for the storage of this substance.

The enthalpy of sublimation and the vapor pressure are important thermodynamic properties of drugs and biologically-active compounds, as knowledge of these values is fundamental in designing and developing industrial and chemical processes. It also contributes to the estimation of the distribution in the environment and for pharmaceutical applications. The rate of sublimation (*k*) obeys the Eyring equation (Equation (1)) from which the enthalpy and entropy of sublimation can be calculated:

(1) $$ln\left(\frac{k}{T}\right)=-\frac{∆H}{RT}+\frac{∆S}{R}$$

In this equation, *k* is the sublimation rate, *T* is the absolute temperature, *∆H* is the enthalpy of sublimation, *∆S* is the entropy of sublimation and *R* is the molar gas constant.

Many methods have been reported for the determination of vapor pressure. The direct determination of the vapor pressure using a manometer is one of them. Probably the most popular is a method known as the Knudsen cell, which involves the measurement of the rate of loss of molecules of the evaporating substance leaving a small orifice in an otherwise closed cell containing the substance of interest [8]. Wiedemann reported the adaptation of a commercial thermobalance to this technique [9]. Goodrum and Seisel have described similar experiments using sealed differential scanning calorimetry (DSC) crucibles with laser drilled holes [10]. Emmeneger and Piccand have described a method for vapor pressure measurements using a standard thermobalance operated under ambient pressure using a special crucible consisting of a bulb containing the sample fitted with a capillary tube [11].

Gückel and co-workers have measured volatilization rates of pesticides at ambient pressure by isothermal thermogravimetry. They proposed a simple though accurate method based on the linear relationship between the logarithm of the sublimation rate and the logarithm of the vapor pressure at the same temperature. Since sublimation and evaporation are zero order processes, the rate of mass loss of a sample under isothermal conditions due to vaporization should be constant provided that the free surface area does not change [12]. Elder and Xie have used the same technique to estimate the vapor pressure of pharmaceutical compounds. They correlated the rate of weight loss with vapor pressure using the behavior of materials of known vapor pressure as standards [13,14]. Price and Hawkins have shown that it is possible to use thermogravimetry to determine the vapor pressure using the Langmuir equation for free evaporation *in vacuo*. In the case of solid substances, it is necessary to melt first to obtain a flat surface. Measurements can be made under isothermal and linear temperature increase conditions using an inert atmosphere instrument purge under ambient pressure [15].

Other methods are based on transpiration techniques. They were used to determine the vapor pressure and then to estimate the enthalpy of sublimation of KBF_4_ [16].

The volatilization of some drugs like nitroglycerin can occur during storage and use of the pharmaceutical dosage form. The reduction of the vapor pressure can be achieved by association of drugs with polymers (e.g., polyvinyl pyrrolidone, polyethylene glycols, microcrystalline cellulose) and β-cyclodextrin [17,18,19].

Cyclodextrins are cyclic oligosaccharides that have been used in many fields due to their ability to encapsulate guest molecules. As an example, they are used to protect against loss by evaporation of volatile compounds in cosmetic products [20]. Pesticides used in the agricultural industry can be complexed with cyclodextrins to reduce their volatility and toxicological effects [21,22,23]. Cyclodextrins are also effective at reducing the volatility of organic pollutants from the chemical industry [24].

The first objective of the present paper is the characterization of the sublimation of G-0 and the quantification of the vapor pressure using an isothermal thermogravimetric method. For this purpose, we applied the method described by Gückel and co-workers [12]. The U.S. Food and Drug Administration (FDA) stipulates that the environmental fate and effects of the drug in the atmosphere should be investigated if its vapor pressure exceeds 1.33 × 10^−5^ Pa [25]. The second objective is to evaluate the effect of the complexation of G-0 with two β-cyclodextrin derivatives, 2-hydroxypropyl-β-cyclodextrin (HP-β-CD) and sulfobutyl ether-β-cyclodextrin (SBE-β-CD), on the sublimation process of the drug. In a previous paper, we described in detail the physicochemical characterization of complexes between G-0 and HP-β-CD or SBE-β-CD. By using a combination of phase solubility analysis, infrared spectroscopy, differential scanning calorimetry, X-ray diffraction, nuclear magnetic resonance spectroscopy and rotating frame nuclear Overhauser effect spectroscopy, we could prove the formation of 1:1 inclusion complexes between G-0 and the two cyclodextrins. The spatial configuration of G-0 inside the cyclodextrin cavity was investigated using molecular modeling studies [26].

## 2. Results and Discussion

### 2.1. Determination of the Purity of G-0 Using DSC

The rate of sublimation of G-0 at a given temperature should be determined by monitoring the rate of weight loss by thermogravimetric analysis (TGA). This method requires the sample to be at least 95% pure and without a substantial amount of residual solvent or other volatile impurities. The drug melts at 73.35 °C, and the purity determined using DSC was 99.95 mol % (see the Supplementary Materials), so the drug clearly satisfied the purity requirements.

### 2.2. Characterization of the Sublimated Product

**Figure 2:**
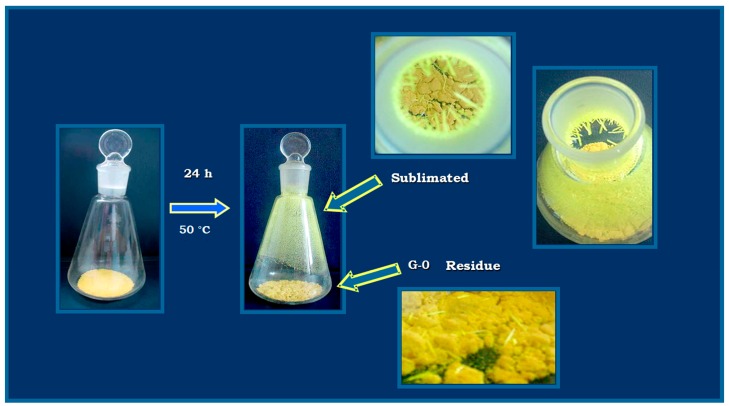
Formation of the sublimated 2-(2-nitrovinyl) furan (G-0).

**Figure 3:**
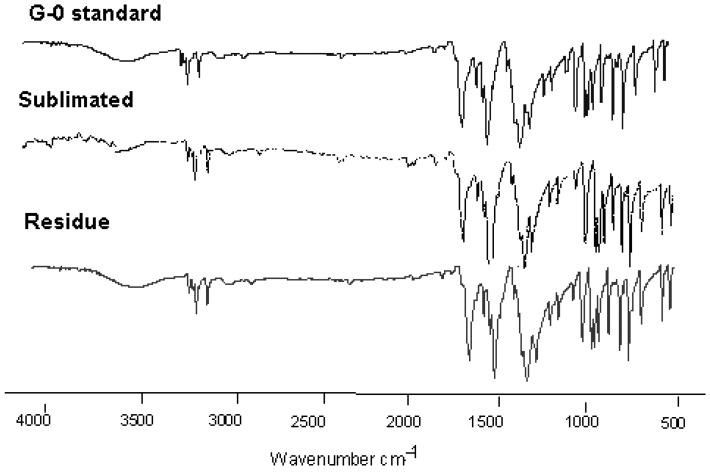
Fourier-transformed-infrared spectra **(**FT-IR, transmission mode) of G-0 standard, sublimated product and residue.

The experiment was performed at 50 °C, since this condition allowed obtaining an adequate amount of sublimated product in a time frame that excluded thermal degradation [27]. Figure 2 shows the solid deposition as needles on the top and upper side of the glass flask in the conditions used in this study. The results obtained from the Fourier-transformed-infrared spectra (FT-IR) (Figure 3) confirmed that the sublimated product is G-0, because all characteristic bands of this substance were present: ν C-H 3116.1 cm^−1^, ν C=C 1633 cm^−1^, ν NO 1570 cm^−1^ and 1322 cm^−1^, ν C-O 1271 cm^−1^, 1150.3 cm^−1^ and 1023.6 cm^−1^. No new bands were observed.

In addition, thin layer chromatography (TLC) showed the same Rf value for the spot corresponding to G-0 in all of the samples (sublimated, residue and reference), and no other spots appeared (see the Supplementary Materials), confirming the identity of the sublimated product.

### 2.3. Sublimation Enthalpy and Vapor Pressure

#### 2.3.1. Sublimation Enthalpy

**Figure 4:**
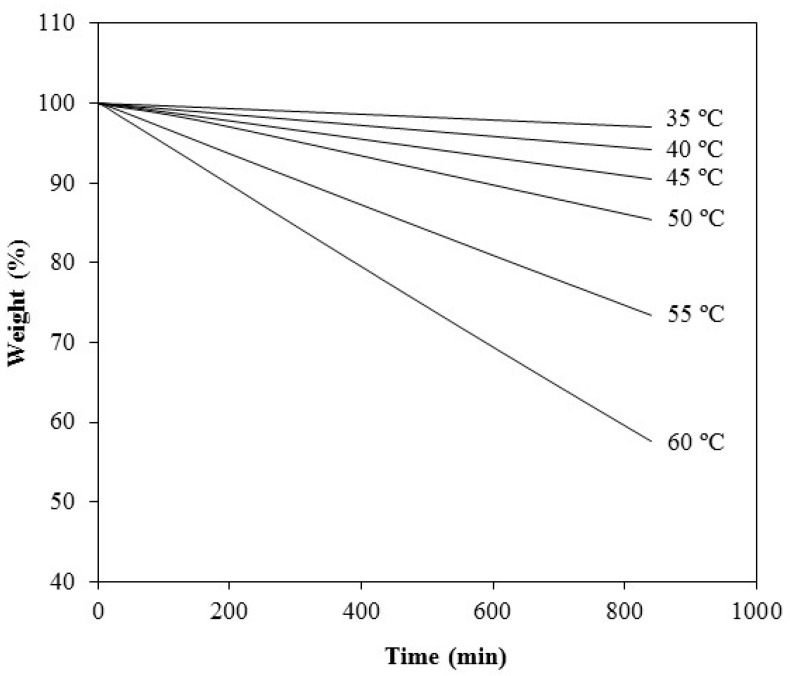
Time-dependent isothermal scans of pure G-0 in the temperature range of 35 to 60 °C.

A representative set of experimental curves displaying the sample weight *vs.* time (Figure 4) shows the weight loss for each storage temperature. It is apparent that the higher the temperature applied, the higher the rate of weight loss obtained. In all cases, the plots were linear (*R*^2^ > 0.9990), indicating that the sublimation followed apparent zero-order kinetics (Table 1).

**Table 1:** Regression parameters obtained from the curves’ G-0 weight loss *versus* time at each temperature.

Temperature (°C)	Linear Regression Equation	Determination Coefficient (*R*^2^)
35	y = −0.0036x + 100.04	0.9998
40	y = −0.0069x + 99.99	1.0000
45	y = −0.0114x + 100.06	1.0000
50	y = −0.0178x + 100.45	0.9991
55	y = −0.0318x + 100.03	1.0000
60	y = −0.0507x + 99.91	0.9999

**Figure 5:**
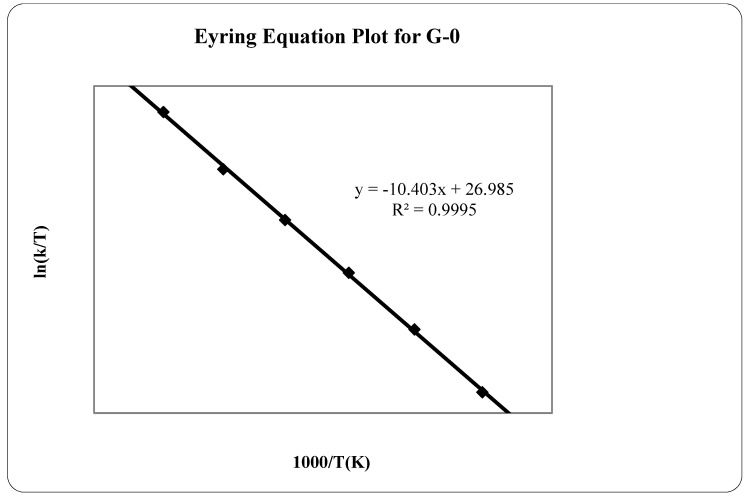
Eyring plot for the G-0 sublimation process.

The fitting of the data to the Eyring equation is shown in Figure 5.

The Eyring equation was used also to calibrate the instrument using the recommended standard benzoic acid, and the sublimation enthalpy determined was 92.5 ± 0.9 kJ·mol^−1^. This value is in agreement with that reported by Wright *et al.* using the same heating rate of 5 °C/min [28].

The sublimation rate of G-0 at each temperature was obtained from the slope of each isothermal TGA curve. The plot of ln(*k*/*T*) *vs.* *1*/*T* gives a straight line with a slope from which the enthalpy of sublimation can be derived, and from the intercept, the entropy of sublimation can be obtained. The sublimation enthalpy and entropy from 35 to 60 °C obtained for G-0 were 86.5 kJ·mol^−1^ and 224.4 J·K^−1^·mol^−1^, respectively. A similar value was obtained for the enthalpy of sublimation of the antibacterial urinary agent, nitroxoline (86.1 kJ·mol^−1^) [29].

#### 2.3.2. Vapor Pressure Estimation

Due to the fact that G-0 undergoes sublimation at relatively low temperatures, the presence of the drug in the atmosphere during drug substance manufacturing needs to be considered. The vapor pressure enables evaluating such an environmental impact. A simple method with sufficient accuracy was proposed by Gückel and coworkers, which is based on the linear relationship between the logarithm of the sublimation rate at a given temperature (*k*) and the logarithm of the vapor pressure (*p*) at this temperature (Equation (2)).

(2) ln(p)=aln(k)+b

Both *a* and *b* are specific constants of a given instrument and a set of experimental conditions and procedures.

This relationship is independent of the material used and the temperature range, but is dependent on the specific instrumental system, experimental conditions and sample containment procedure.

In this study, benzoic acid was used as a reference material to calibrate the TGA instrument and experimental conditions, because its vapor pressure has been accurately determined at different temperatures using multiple methods [9,13].

The sublimation rate for benzoic acid from 40 to 70 °C was determined using an identical procedure and experimental configuration as for G-0, and the data are shown in Table 2.

**Table 2:** Experimental sublimation rate obtained using the TGA system and reported vapor pressure of benzoic acid in the temperature range from 40 to 70 °C.

Temperature (°C)	Sublimation Rate (µg/min)	Vapor Pressure (Pa)
40	0.0796	0.910
45	0.1507	1.546
50	0.2909	2.582
55	0.4649	4.245
65	1.2250	10.985
70	2.0981	17.306

The vapor pressures of benzoic acid at the corresponding temperatures used were obtained from [9].

**Figure 6:**
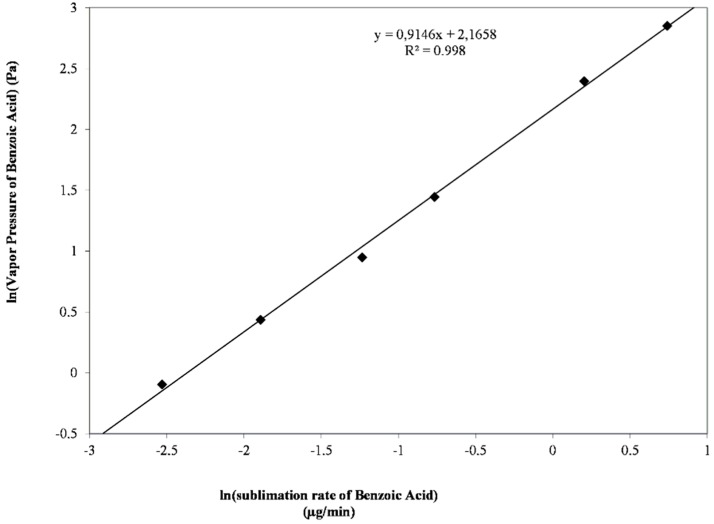
Linear relationship between the ln (sublimation rate) and ln (vapor pressure) of benzoic acid (reported in the literature).

The logarithm of the experimental sublimation rates of benzoic acid was plotted against the reported vapor pressure at each temperature (Figure 6).

Equation (2) was fitted to the data, and both constants, *a* and *b*, were obtained with an *R*^2^ = 0.998, as shown in Equation (3).

(3) ln(p)=0.9146ln(k)+2,1658

Using the equation above and the measured sublimation rate, the vapor pressure of G-0 was calculated at each temperature as shown in Table 3.

The sublimation rate at room temperature of G-0 could be determined by extrapolation from measured rates of sublimation at higher temperatures using the Eyring equation. This calculation assumed that the enthalpy and entropy of sublimation were constant throughout the temperature range as long as G-0 remained in the same physical form. The sublimation rate at 25 °C calculated was 0.1078 µg/min, which produced a vapor pressure of 1.137 Pa. Taking into account the criterion established by the FDA for the vapor pressure (1.33 × 10^−5^ Pa) [25], the value obtained for the drug was higher than the limit, suggesting that the environmental fate and effects of the drug in the atmosphere should be investigated. Ideally, future formulations must try to minimize this physical stability problem.

**Table 3:** Sublimation rate and calculated vapor pressure for G-0.

Temperature (°C)	Sublimation Rate (µg/min)	Calculated Vapor Pressure (Pa)
35	0.3401	3.252
40	0.6146	5.588
45	1.0503	9.122
50	1.7310	14.405
55	2.7983	22.352
60	4.8075	36.667

### 2.4. Evaluation of the Influence of Complexation with Cyclodextrins on the Sublimation Process

In order to accurately interpret the TGA curves for analysis of the sublimation of G-0 from the G-0-CD complexes, one has to know the residual water content of the complexes, which will be present as a consequence of the presence of water in the pure CDs, but also as a consequence of the preparation methods.

The results showed comparable water contents for both cyclodextrins and the freeze-dried complex using HP-β-CD. The water content of the freeze-dried complex with SBE-β-CD was slightly higher (Table 4). These results will be taking into account to correctly quantify the sublimation experiments.

**Table 4:** Karl Fischer titration (KFT) for the water content of freeze-dried (FD) complexes and cyclodextrins. SBE-β-CD, sulfobutyl ether-β-cyclodextrin; HP-β-CD, 2-hydroxypropyl-β-cyclodextrin.

Sample	Water Content (%) ^a^
SBE-β-CD	6.38 ± 0.87
HP-β-CD	6.40 ± 0.02
FD G-0/SBE-β-CD	8.33 ± 0.36
FD G-0/HP-β-CD	5.81 ± 0.27

^a^ Mean ± standard deviation.

#### 2.4.1. HP-β-CD Inclusion Complexes

**Figure 7:**
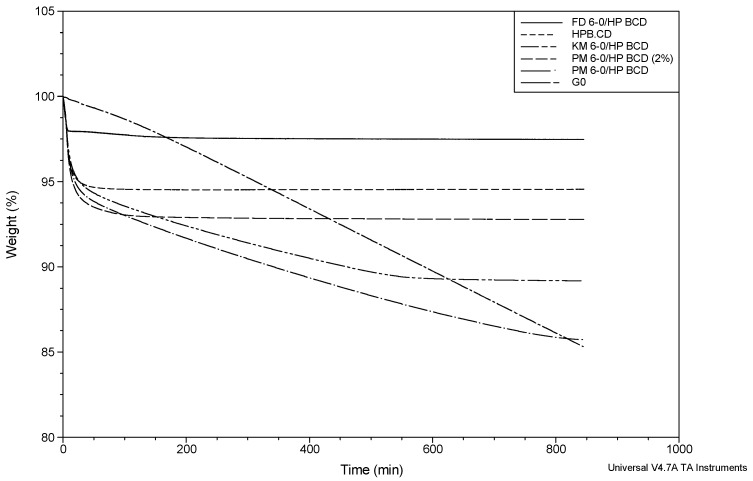
Isothermal time-dependent TGA curves of: pure G-0 (broken dash), HP-β-CD (short dash), physical mixture (PM) G-0/HP-β-CD (2%) (dash), physical mixture (PM) G-0/HP-β-CD (8.8%) (dash dot), kneaded mixture (KM) G-0/HP-β-CD (8.8%) (broken double dash) and freeze-dried (FD) G-0/HP-β-CD (2%) (solid line).

TGA is a method of thermal analysis in which changes in physical and chemical properties of materials are measured as a function of increasing temperature or as a function of time [30,31]. The isothermal TGA curve of HP-β-CD showed a total weight loss of 5.77% ± 0.46%, occurring mainly in the first 30 min. From this time and until 200 min, the weight loss was much lower, and also, the slope obtained was close to zero from 200 min to the end of the run (Table 5 and Figure 7). The weight loss is attributable to the removal of the water included in the raw material, since the decomposition of this cyclodextrin is reported at temperatures higher than 300 °C [32].

**Table 5:** Mean and standard deviation of weight loss and sublimation rate (slope) of different cyclodextrin systems with and without G-0, obtained from TGA curves. PM = physical mixture, KM = kneaded mixture, FD = freeze-dried mixture.

System	Total Weight Loss (%)	Weight Loss 1 (%)	Weight Loss 2 (%)	Slope 1 (%/min)	Slope 2 (%/min)
HP-β-CD	5.77 ± 0.46	5.35 ± 0.35 ^a^	0.42 ± 0.06 ^b^	−0.20 ± 0.03 ^a^	−0.14 × 10^−2^ ± 0.18 × 10^−3^ ^b^
PM G-0/HP-β-CD (2%)	7.47 ± 0.35	6.22 ± 0.28 ^a^	1.19 ± 0.15 ^b^	−0.20 ± 0.66 × 10^−2^ ^a^	−0.55 × 10^−2^ ± 1.01 × 10^−3^ ^b^
PM G-0/HP-β-CD (8.8%)	13.88 ± 0.36	4.86 ± 0.28 ^a^	9.01 ± 0.08 ^e^	−0.29 ^a^	−1.1 × 10^−2^ ± 0.12 × 10^−3^ ^e^
KM G-0/HP-β-CD	10.88 ± 0.21	5.65 ± 0.67 ^a^	5.03 ± 0.36 ^c^	−0.19 ^a^	−0.95 × 10^−2^ ± 0.56 × 10^−3^ ^c^
FD G-0/HP-β-CD	4.36 ± 1.58	3.85 ± 1.55 ^a^	0.41 ± 0.04 ^b^	−0.55 ^a^	−0.23 × 10^−2^ ± 0.48 × 10^−4^ ^b^
SBE-β-CD	6.92 ± 0.55	4.61 ± 0.54 ^a^	2.09 ± 0.02 ^b^	−0.22 ± 0.28 × 10^−1^ ^a^	−0.70 × 10^−2^ ± 0.30 × 10^−4^ ^b^
PM G-0/SBE-β-CD (1.7%)	9.67 ± 0.22	6.60 ± 0.14 ^a^	2.89 ± 0.08 ^b^	−0.21 ± 0.21 × 10^−3^ ^a^	−1.37 × 10^−2^ ± 0.54 × 10^−3^ ^b^
PM G-0/SBE-β-CD (7%)	12.18 ± 0.06	5.16 ± 0.20 ^a^	6.90 ± 0.08 ^d^	−0.17 ± 0.41 × 10^−1^ ^a^	−1.61 × 10^−2^ ± 0.37 × 10^−2^ ^d^
KM G-0/SBE-β-CD	11.85 ± 0.80	6.88 ± 1.17 ^a^	4.83 ± 0.36 ^c^	−0.26 ± 0.98 × 10^−1^ ^a^	−1.54 × 10^−2^ ± 0.12 × 10^−2^ ^c^
FD G-0/SBE-β-CD	5.84 ± 2.40	5.46 ± 2.87 ^a^	0.33 ± 0.13 ^b^	−0.28 ± 0.12 ^a^	−0.13 × 10^−2^ ± 0.02 × 10^−2^ ^b^

^a^ From 0 to 30 min; ^b^ from 30 to 200 min; ^c^ from 30 to 450 min; ^d^ from 30 to 600 min; ^e^ from 30 to 840 min.

Two physical mixtures (PM) of G-0/HP-β-CD were prepared with 2% and 8.8% of G0in order to compare these samples with the corresponding freeze-dried and kneaded complexes.

The curve related to the PM G-0/HP-β-CD prepared with 2% of API showed a similar shape to that of the HP-β-CD curve (Figure 7), which can be expected taking the low content of G-0 in the sample into account. However, the total weight loss (7.47% ± 0.35%) was higher compared to that obtained for pure HP-β-CD, and a higher slope from 30 to 200 min was obtained (Table 5). In the absence of additional water in the physical mixtures, these increased values are related to the sublimation of G-0.

The curve corresponding to the PM G-0/HP-β-CD prepared with 8.8% of the API exhibited a noticeable change in its shape compared to the systems described above. The total weight loss was 13.88% ± 0.36%, and a remarkable increase in the slope from 30 min to the end of the run was noticed as a consequence of the sublimation of the drug (Table 5 and Figure 7). Previous X-ray powder diffraction (XRPD) studies carried out over physical mixtures of G-0 with these cyclodextrins showed some characteristic peaks attributed to the uncomplexed drug. Furthermore, the melting peak of G-0 appeared in the DSC thermograms, pointing to free drug in those physical mixtures [26].

The inclusion complexes G-0/HP-β-CD obtained using the freeze-dried method were previously confirmed by nuclear magnetic resonance studies. In this experiment, the FD complex showed a similar thermal behavior as that of pure HP-β-CD. However, differences were detected, associated with a higher slope of weight loss during the first 20 min. This result is most likely caused by the nature of this kind of complex, characterized by a very porous and amorphous matrix, which was previously characterized using XRPD [26], which allows the easy release of water. The average of the weight loss obtained was very similar to that obtained for the corresponding free cyclodextrin and also similar to that measured using Karl Fischer titration (KFT). If these results are compared to those obtained for the physical mixture with 2% of G-0 (the same proportion of API on the freeze-dried complexes), we can conclude that freeze-drying, as a method to prepare the complexes with HP-β-CD, significantly reduces the sublimation rate of G-0 and also the weight loss (see Figure 7 and Table 5). Similar behavior was reported for the 2-undecanone/α-CD inclusion complex and also for the citral/monochlorotriazinyl-β-CD freeze-dried complex [33,34].

The curve related to the G-0/HP-β-CD complex prepared by kneading was similar to that obtained for the physical mixture prepared at 8.8% of API, but only until 450 min. From this point on to the end, the slope of the curve was flat (Figure 7), which suggests that the sublimation process stopped, opposite the case of the physical mixture where the sublimation seems to continue. The total weight loss (10.88% ± 0.21%) was lower than that obtained for the corresponding physical mixture (Table 5). These findings suggest that the drug is only partially involved in the inclusion complex, and only that part which is complexed is protected from sublimation. The amount of API prone to sublimation can be related to the free drug in the complex.

#### 2.4.2. SBE-β-CD Inclusion Complexes

The curve corresponding to SBE-β-CD had a similar behavior to that recorded for HP-β-CD. However, HP-β-CD seems to release the water faster than SBE-β-CD, which is supported by the higher value of the slope obtained during the first 30 min for the hydroxy-propoxylated derivative (Table 5). The total weight loss (6.92% ± 0.55%) obtained due to the water evaporation was very similar to that calculated by KFT.

Two physical mixtures G-0/SBE-β-CD were prepared with 1.7% and 7.0% of API for comparative purposes with the corresponding freeze-dried and kneaded complexes.

**Figure 8:**
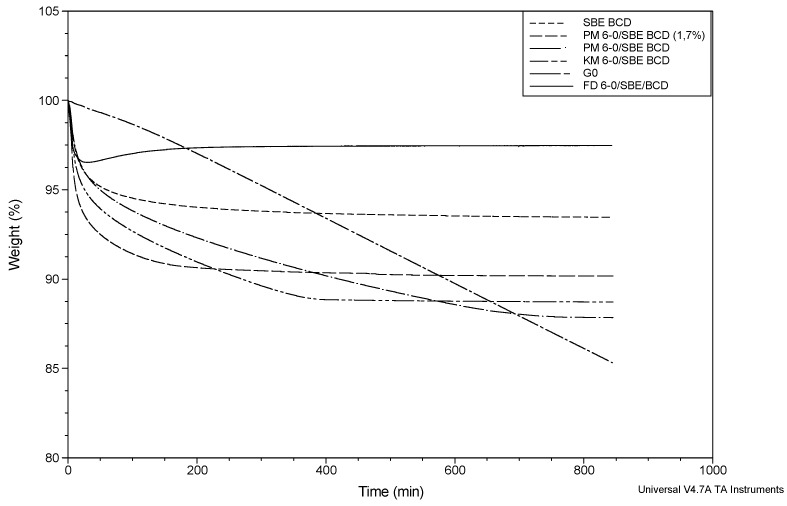
Isothermal time-dependent TGA curves of: pure G-0 (broken dash), SBE-β-CD (short dash), physical mixture (PM) G-0/SBE-β-CD (1.7%) (dash), physical mixture (PM)G-0/SBE-β-CD (7%) (dash dot), kneaded mixture (KM) G-0/SBE-β-CD (7%) (broken double dash) and freeze-dried (FD) G-0/SBE-β-CD (1.7%) (solid line).

The curve for the PM G-0/SBE-β-CD prepared with 1.7% exhibited a similar behavior as that obtained for the physical mixture at 2% of G-0 with HP-β-CD (Figure 8).

In this case, a total weight loss of 9.67% ± 0.22% was detected, which was higher than that obtained for pure SBE-β-CD. This suggests, similar to the system with HP-β-CD, that the additional mass loss is due to sublimation of G-0 from the physical mixture.

The analysis of the curve corresponding to the physical mixture prepared with 7% of API showed a total weight loss of 12.18% ± 0.06%. The pattern was very similar to that obtained for the physical mixture of HP-β-CD with 8.8% until 600 min, and from that point on, the weight loss is largely reduced. This finding can be related to the lower content of G-0 in the physical mixture with SBE-β-CD (7%) compared to 8.8% with HP-β-CD, so the amount of G-0 available for sublimation is also lower, and the process can finish earlier.

TGA curves of the freeze-dried complexes with SBE-β-CD, in general, exhibited the same shape as the curves of the freeze-dried complexes with HP-β-CD. The average of the weight loss calculated was 5.84% ± 2.40% (occurring mainly in the first 20 min), which was similar to that obtained for SBE-β-CD and slightly lower than the previous water content determination for this complex using KFT. This titration allows one to obtain the total amount of water contained in the sample, and the isothermal TGA allowed estimating the amount of water loss at the temperature of 50 °C, so probably not all of the solvent is removed from this freeze-dried complex. From 20 min to the end, the curve becomes flat, indicating that the weight loss stopped, so no sublimation process was observed at this stage (Table 5 and Figure 8). The comparison between the results obtained for this kind of complex and the corresponding physical mixture at the same proportion of API demonstrated that the sublimation of the drug is clearly limited in the complex.

Finally, the TGA curves of the G-0/SBE-β-CD kneaded complexes showed a total weight loss of 11.85% ± 0.80%. A change in the slope of the curve from 400 min until the end of the run was noticeable (the curve becomes flat), indicating that the sublimation process stopped before that of the physical mixture (Table 5 and Figure 8). These differences indicate that the drug that was not included in the cyclodextrin could sublimate, but not the amount that was inside the hydrophobic cavity of the excipient.

The main differences encountered between the TGA curves for freeze-dried and kneaded complexes can be related to the manufacturing procedure of the complexes [26]. Before equilibration, the non-dissolved G-0 is removed by filtration from the suspensions of drug in cyclodextrin solution prior to the freeze-drying process. In the second method, the entire drug powder is added to the cyclodextrin paste, and this mixture is then kneaded in a mortar. Hence, the amount of G-0 that is not part of the inclusion complex remains in the final kneaded product, and its sublimation is registered during the TGA experiment.

## 3. Experimental Section

### 3.1. Materials and Reagents

2-(2-Nitrovinyl) furan (G-0) was synthetized according to the method reported [1] and was kindly supplied by the Centro de Bioactivos Químicos (Villa Clara, Cuba).

Benzoic acid, (certified standard titrimetric substance, 100.022% ± 0.070% purity) was purchased from Fluka, (Buchs, Switzerland) and disodium tartrate dihydrate dibasic from Merck (Darmstadt, Germany).

2-Hydroxypropyl-β-cyclodextrin (HP-β-CD) was purchased from JANSSEN Pharmaceutica NV. (Beerse, Belgium) and sulfobutyl ether-β-cyclodextrin (SBE-β-CD) from CyDex Inc. (Lenexa, KT, USA).

All other solvents and reagents used in this study were of analytical grade.

### 3.2. Determination of the Purity of G-0 Using DSC

A DSC Q2000 (TA-Instruments, Brussels, Belgium) was used to calculate the purity of the sample under study in the sublimation experiment. Indium and *n*-octadecane were used as standard materials to calibrate the enthalpy and temperature. Once the calibration was performed, *ca.* 3 mg of the drug were weighed in aluminum pans. The heating rate used was 2 °C/min from 10 to 100 °C. The purity analysis was carried out using the resident software (Universal Analysis, TA-Instruments, Brussels, Belgium).

### 3.3. Characterization of the Sublimated Product

Approximately 4.0 g (accurately weighed) of G-0 was added to a 250-mL Erlenmeyer flask, and the system was sealed using a glass cap and parafilm. The bottom of the flask was immersed in a Heildolph thermoregulated water bath at 50 °C for 24 h and protected from light using aluminum foil. Finally, the solid deposited on the cap and the top of the flask was collected and further characterized using FT-IR and TLC.

#### 3.3.1. Fourier-Transformed-Infrared Spectroscopy

FT-IR analysis was performed on samples prepared using the potassium bromide disc method. Approximately 0.5 to 1.0 mg of each sample were weighed (Sartorius Analytical Balance, Sartorius, Boutersem, Belgium) and mixed with 200 mg of previously dried KBr in an agate mortar. The samples were compressed using a hydraulic press. The spectra were recorded in a Perkin Elmer Infrared Spectrometer (Spectrum RX/FTIR System, Perkin Elmer, Waltham, MA, USA) in the spectral region of 4000 to 500 cm^−1^. The instrument was calibrated previously using a polystyrene calibration film.

#### 3.3.2. Thin Layer Chromatography

A sample of G-0 reference standard (*ca.* 5 mg accurately weighed) was transferred to a 10-mL volumetric flask, and the volume was made up with dichloromethane (Solution A). The same procedure was applied to prepare the solutions containing the residue (in the bottom of the Erlenmeyer) and the sublimated product (Solutions B and C, respectively).

A chromatographic chamber was saturated with a mobile phase consisting of carbon tetrachloride and chloroform (9:1, *v*/*v*). Solutions A, B and C were applied (10 µL) to a Silica gel 60 thin layer plate, at a distance of 1.5 cm from the lower edge and 1.5 cm from each other. Once the solvent was evaporated, the thin layer plate was placed in the chromatographic chamber until the eluent achieved a distance of 1.5 cm from the upper edge. Then, it was removed from the chamber, dried at room temperature, and spots were detected using iodine vapor [35].

### 3.4. Determination of Sublimation Enthalpy and Vapor Pressure of G-0

The sublimation rates of G-0 at a given temperature were determined using isothermal TGA (Q600, TA-Instruments, Brussels, Belgium). Data acquisition and analysis were performed using Universal Analysis (TA-Instruments, Brussels, Belgium). A constant nitrogen gas purge of 50 mL·min^−1^ was used during the experiments. The furnace was heated at 5 °C/min to the desired temperature, and then, it was kept isothermal for 840 min in each run. The sample amount was *ca.* 10 mg (accurately weighed). At least 30% weight loss was monitored for each run to allow an accurate determination of the rate of weight change. The sublimation rate at each temperature was obtained using the slope of the linear regression model. The temperatures used for the experiments were 35, 40, 45, 50, 55 and 60 °C (all below the melting point of the drug).

Benzoic acid was used as a reference standard for the vapor pressure estimation by TGA. The isothermal sublimation rates of benzoic acid were determined using the same method and equipment as for G-0, in the temperature range of 40 to 70 °C.

### 3.5. Evaluation of the Influence of Complexation with Cyclodextrins on the Sublimation Process

#### 3.5.1. Karl Fischer Titration for Water Content Determination of the Cyclodextrin Complexes and Pure Cyclodextrins

KFT was performed using a 701 KF Titrino, Metrohm, (Herisau, Switzerland). *ca.* 100 mg of disodium tartrate dihydrate dibasic (puriss, p.a) was used as the standard for the water content assay; methanol was used as the solvent. The experiment was done in triplicate, and the mean value and standard deviation were calculated. The water content assay for cyclodextrin complexes and pure cyclodextrins was performed with *ca.* 200 mg of product.

#### 3.5.2. Isothermal TGA of the Cyclodextrin Complexes

Complexes of cyclodextrins with G-0 were prepared by freeze drying and kneading according to previously-reported methods [26]. Four physical mixtures were prepared additionally in the same G-0-CD ratio as in both complexes: (1) 1.7% G-0 in G-0/SBE-β-CD physical mixture; (2) 2.0% in G-0/HP-β-CD physical mixture, in order to compare to the sublimation rate and weight loss of the freeze-dried complexes; (3) 7.0% G-0 in G-0/SBE-β-CD physical mixture; and (4) 8.8% in G-0/HP-β-CD physical mixture, to compare to the sublimation rate and weight loss of kneaded complexes.

Isothermal TGA experiments were conducted using thin samples (thickness of *ca*. 0.2 to 0.4 mm). The experiments were done using the same instrument as described above. The samples were tested in open aluminum sample pans under nitrogen atmosphere with a flow rate of 50 mL·min^−1^ and a temperature of 50 °C, for 840 min.

In order to evaluate the sublimation of G-0 from the complexes, the slopes of the linear parts of the curves and the weight loss during the process were obtained.

## 4. Conclusions

The sublimation of G-0 in the temperature range of study followed apparent zero-order kinetics. The sublimation enthalpy from 35 to 60 °C obtained using the Eyring equation was 86.5 kJ·mol^−1^. The Gückel method allowed estimating the sublimation rate and the vapor pressure of the drug at 25 °C. The values obtained were 0.1078 µg/min and 1.137 Pa.

Our results indicate that the formation of inclusion complexes with CD derivatives can reduce the sublimation of G-0. Hence, the method of inclusion complex preparation seems to have a clear influence on the degree of protection against sublimation.

## Supplementary Materials

Supplementary materials can be accessed at: http://www.mdpi.com/1420-3049/20/08/15175/s1.
