# Supplementary material for: Characterization of the Sublimation and Vapor Pressure of 2-(2-Nitrovinyl) Furan (G-0) Using Thermogravimetric Analysis: Effects of Complexation with Cyclodextrins
Source: Molecules. 2015 Aug 19;20(8):15175–91. doi: 10.3390/molecules200815175 (PMC6332437; doi:10.3390/molecules200815175)
Supplement: Supplementary file 1 [file molecules-20-15175-s001.pdf]

Supplementary Material

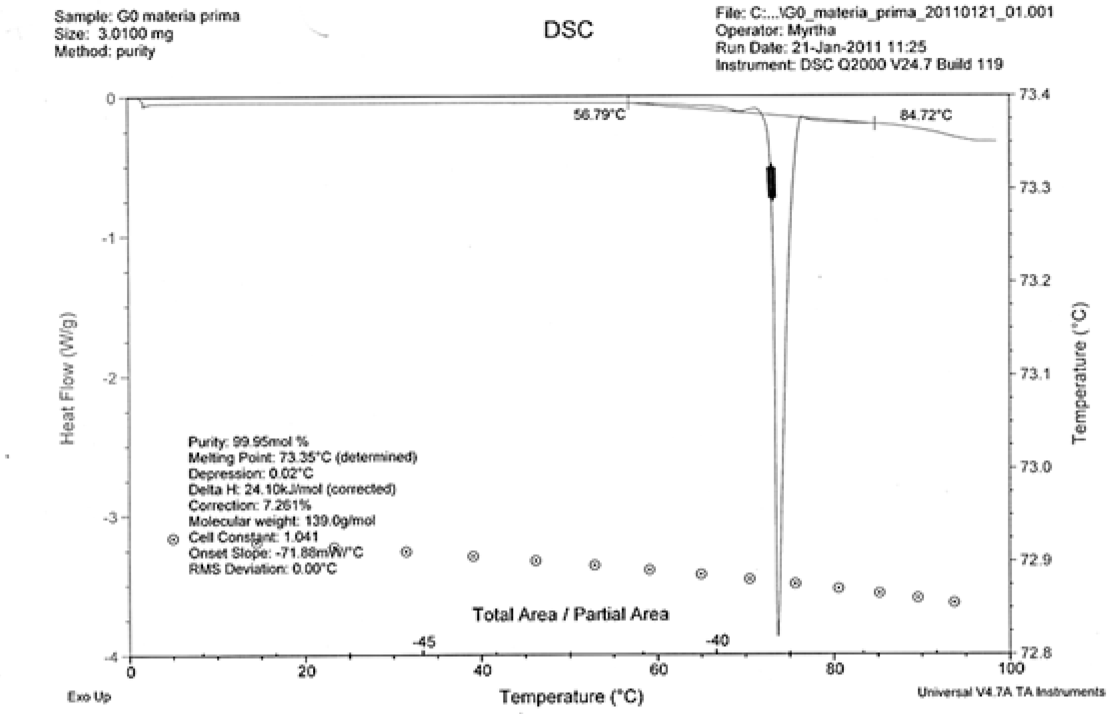

Figure S1. DSC obtained for the purity analysis of G-0.

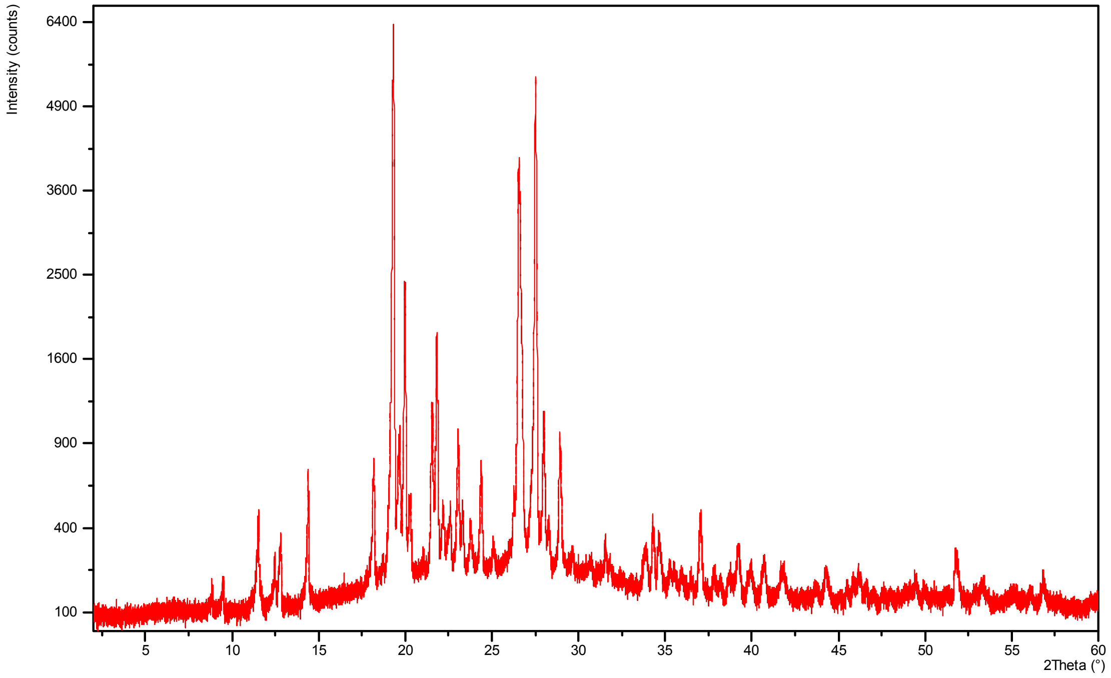

Figure S2. X-ray diffractogram of pure G-0.

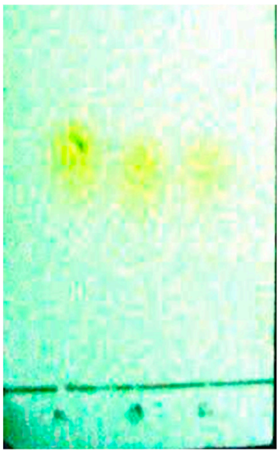

A B C

Figure S3. Thin Layer Chromatography obtained for, A: G-0, sample reference; B: Residue; C: Sublimated product.
